# The Role of Biomechanical Regulation in Physiological and Pathological Pregnancy: From Mechanotransduction to Clinical Implications

**DOI:** 10.7150/ijbs.134599

**Published:** 2026-08-12

**Authors:** Tingting Wang, Wanru Chen, Huixia Yang, Jie Yan

**Affiliations:** Department of Obstetrics and Gynecology, Peking University First Hospital, Beijing, 100034, China.

**Keywords:** maternal-fetal interface, biomechanics, extracellular matrix, mechanotransduction, pregnancy complications

## Abstract

Pregnancy is a highly coordinated process that relies on the precise regulation of the extracellular matrix, cells, and various molecules at the maternal-fetal interface. As the fetus grows and amniotic fluid increases, the uterus undergoes adaptive remodeling, a process in which biomechanics plays an indispensable role. This review aims to systematically explore the biomechanical characteristics of the uterus and specifically elucidate the crucial regulatory roles of mechanical signals such as stiffness, shear stress, tension, and compression force in both physiological and pathological pregnancies. We focus on the remodeling of the extracellular matrix, the mechanical property changes of the maternal-fetal interface, and the associated mechanotransduction pathways, such as PIEZO1, integrins, and YAP/TAZ. Finally, we summarize the cutting-edge technologies used to investigate uterine biomechanics and discuss the clinical translational potential of targeting mechanical signaling pathways as novel diagnostic and therapeutic strategies for pregnancy complications. This review provides a comprehensive analysis of how biomechanical cues regulate uterine function at the molecular, cellular, and tissue levels.

## Introduction

Pregnancy is a highly coordinated biological process involving embryo implantation, placental development, and fetal growth within a dynamically changing maternal environment. The success of this process depends on the precise regulation of cellular, molecular, and tissue-level processes within the maternal-fetal microenvironment. Traditional research on pregnancy mainly focused on genetic and biochemical regulation. Hormonal regulation 1, 2, immune tolerance 3, and metabolic disorders 4 have been widely recognized as key contributors.

The maternal-fetal interface is a complex system composed of various components, including decidual stromal cells (dS), trophoblasts, immune cells, and the extracellular matrix (ECM) (5). These components do not exist independently but are involved in cell activities and intercellular communication through numerous biochemical signals. The role of biomechanics in this process has recently attracted attention. Biomechanics primarily applies mechanics principles to biological systems, providing a framework for understanding how mechanical properties influence cellular behavior and tissue function 5. Animal and *in vitro* models have indicated that such mechanical cues may modulate processes such as vascular remodeling 6 and trophoblast behavior 7. More importantly, mechanical abnormalities may lead to impaired placental development, contributing to complications such as placenta accreta spectrum (PAS) 8 and preeclampsia (PE) 9. Nevertheless, the extent to which these mechanisms operate in human pregnancy and how they integrate with known biochemical pathways remains unclear.

Several reviews have explored uterine biomechanics 10 and mechanotransduction at the maternal-fetal interface in isolation 11. Given the growing but heterogeneous body of evidence, a systematic and critical review is needed to clarify the mechanical characteristics of the maternal-fetal interface and its spatiotemporal dynamics. This review aims to follow the “mechanics-structure-signal-function-disease” axis as the main thread and to conduct a hierarchical, comprehensive analysis of the existing evidence. Specifically, we discuss how mechanical signals shape tissue organization, are perceived by cells, and are transformed into cellular responses, ultimately leading to physiological adaptation or pathological progression. We also evaluate current biomechanics-related tools based on purpose and clinical maturity. Moreover, we discuss their translational potential, as well as the prospects and limitations of targeted mechanical drug application in pregnancy complications.

## Uterine Biomechanical Framework: From Tissue Mechanics to Mechanotransduction

### Uterine Structure and Biomechanical Properties

The uterus is an organ composed of muscle tissue. The uterine wall is composed of the endometrium, the myometrium, and the perimetrium. Each layer contributes distinct mechanical functions. The endometrium can provide a contact interface for embryo implantation, the myometrium can generate contractile forces, and the perimetrium can preserve structural integrity. This functional differentiation suggests that pregnancy-adaptive remodeling may exhibit layer-specific characteristics. As pregnancy progresses, the uterine cavity expands significantly by 500 to 1000 times 12, accompanied by pronounced structural remodeling of the uterine wall. Early pregnancy is dominated by tissue growth, whereas later stages involve progressive stretching and thinning of the uterine wall 13. Imaging-based studies on humans have demonstrated specific changes in myometrium thickness across different regions. For example, the thickness of the myometrium in the lower uterine segment decreases significantly with advancing gestational age, whereas the myometrium at the placental attachment site becomes markedly thicker 14. During the second and third trimesters, the fundal myometrium is the thinnest 15. In the intrapartum period, the myometrium first thins symmetrically and then undergoes a rebound thickening 16. These spatial differences are not randomly distributed. Instead, they may reflect the structural adaptation of the uterus to meet region-specific mechanical load requirements, thereby influencing the load distribution and contractile function.

**Figure 1:**
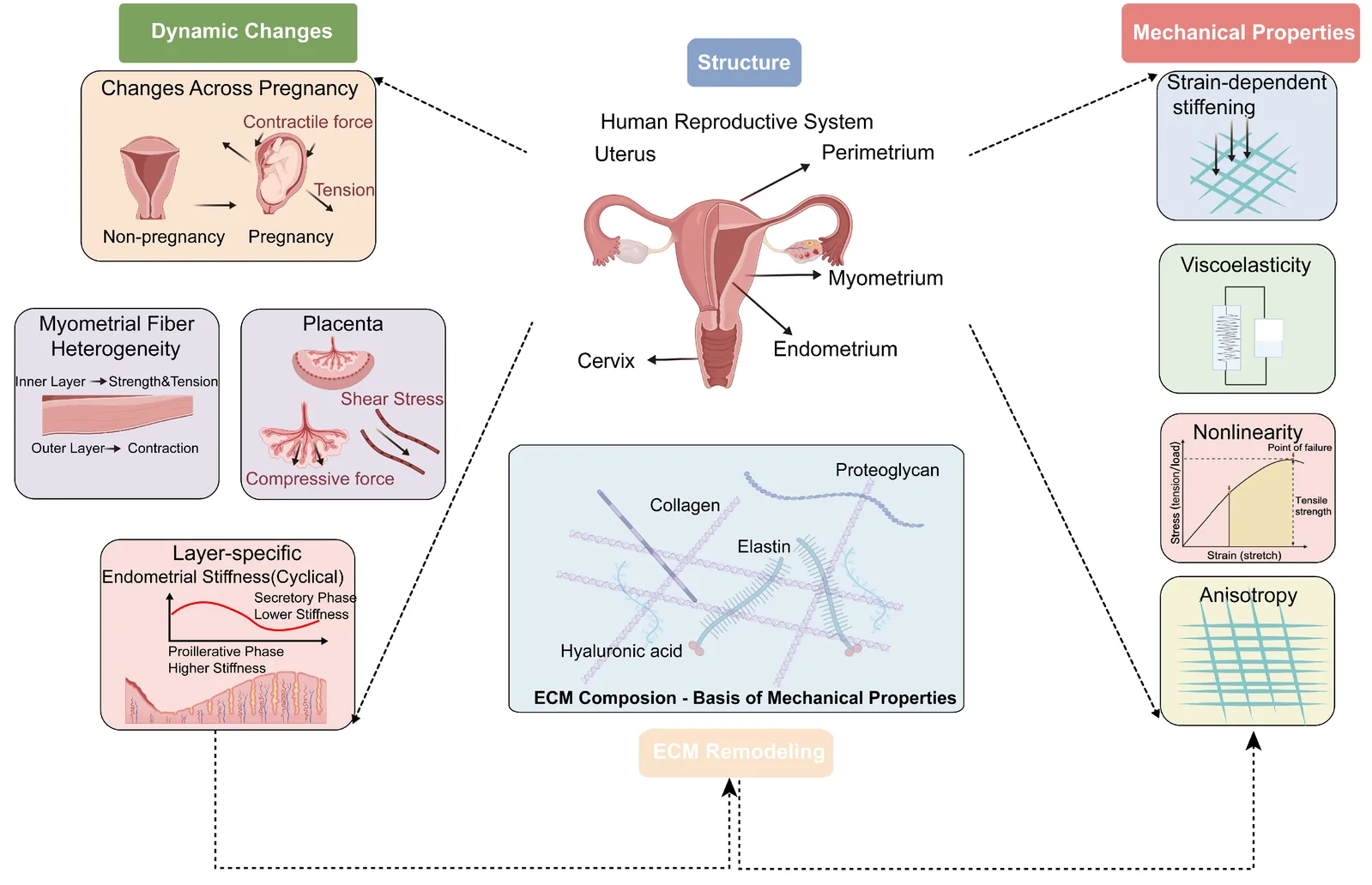
** Structure and mechanical characteristics of the uterus.** The uterus is a complex hollow organ whose wall is mainly composed of three layers: the endometrium, myometrium, and perimetrium. The myometrium consists of smooth muscle, with an outer longitudinal layer ensuring contractile function and an inner circular layer primarily responsible for integrity. During the menstrual cycle, the mechanical properties of the uterus change periodically, exhibiting decreased stiffness during the secretory phase. Furthermore, the uterus is continuously subjected to external multiscale forces, including uterine wall tension caused by fetal growth, compressive forces from villous growth, and shear stress. Changes in extracellular matrix (ECM) composition and structure confer typical soft tissue mechanical properties on the uterus: strain-dependent stiffening, viscoelasticity, anisotropy, and nonlinear characteristics.

Uterine tissue exhibits typical biomechanical characteristics of soft biological materials, including anisotropy, nonlinearity, frequency dependence, viscoelasticity, and strain-dependent stiffening **(Figure 1)**. These properties collectively reflect the way the uterus responds to mechanical loads to accommodate the dynamic demands of pregnancy. Specifically, the diffusion anisotropy of the uterus is related to its ordered fiber structure 17, 18. Experimental studies have characterized uterine mechanical behavior under both compressive and tensile loads. The uterine tissue displays relatively low resistance to compression but exhibits strain-stiffening behavior under tension, in which its resistance to deformation increases with increasing strain 19, 20. Within the frequency range of 0.1 to 100 Hz, the modulus of uterine tissue shows greater frequency dependence, consistent with its viscoelastic behavior 21. Although these observations have been primarily from *in vitro* mechanical tests, they provide key parameters for quantifying the integrity and functional reserve of the uterine structure.

Of note, uterine biomechanical properties are not constant, as shown in** Table 1**. *Ex vivo* studies suggest that during pregnancy, the passive compliance and extensibility of uterine tissue gradually increase to accommodate the growth needs of the fetus. One study found no effect of parity, age, or menstrual cycle stage on uterine mechanical properties 22. In contrast, *in vivo* imaging studies indicate that endometrial stiffness is higher during the proliferative phase than during the secretory phase 23. These inconsistent findings may be attributed to methodological heterogeneity and structural heterogeneity across the uterine layers (periodic hormonal effects may act on the endometrium rather than the myometrium), so these results are not always directly comparable. Near the implantation window, the uterus adjusts its mechanical properties to prepare for subsequent embryo implantation. Understandably, the tension in the uterine wall increases as pregnancy progresses, consistent with the continuously increasing mechanical demands imposed by fetal development 24. During delivery, the mechanical behavior of the uterus is characterized by nonlinear stress-strain responses and coordinated contraction activity, which may be influenced by tissue structure and loading conditions 25. On one hand, uterine fibers exhibit structural heterogeneity. Dense muscle fibers are arranged to withstand tension, while the longer outer muscle layer fibers are responsible for contraction 26. On the other hand, the uterine smooth muscle undergoes passive stress relaxation at a slower rate, which further maintains the stability of uterine wall tension and promotes the formation of the lower uterine segment 27. Due to ethical and technical limitations in obtaining human samples throughout pregnancy, animal models provide valuable complementary insights. Studies in rhesus monkeys have suggested the presence of spatial gradients in mechanical properties across uterine layers. Specifically, the stiffness from the endometrium to the perimetrium increases gradually. The viscoelastic ratio of the endometrium is higher, while its permeability and diffusivity are lower than those of the myometrium and perimetrium 10. This is slightly different from the increase in uterine decidua stiffness observed during human pregnancy, possibly due to species differences 28. Additionally, the stiffness of uterine tissues under pathological conditions also varies. Uterine fibroids are stiffer than normal tissues, while malignant tissues are the opposite. This suggests that there may also be biomechanical differences between different uterine pathologies 29. Interspecies differences should be considered when extrapolating these findings to human pregnancy.

At the organ level, the uterus does not function in isolation, but forms a mechanically coupled system with the cervix and fetal membranes. Its moderate stiffness and ability to expand are crucial for accommodating fetal growth and transmitting mechanical loads 30. Finite element models indicate that the cervix, characterized by a complex three-dimensional stress state, contributes to load redistribution among the uterus, fetal membranes, and itself, with its stretching depending on geometric morphology and material properties 31, 32. These biomechanical properties ensure the uterine structural stability and adaptability throughout gestation.

### Mechanotransduction Pathways in the Uterus

At the microscopic level, the mechanical behavior of the uterus is related to the composition of the ECM. The uterine ECM is primarily composed of type I, III, and V collagens, laminin, fibronectin, and proteoglycans, which together form a fibrous network that supports the tissue structure 33. Importantly, beyond composition, the organization, arrangement, and cross-linking density of collagen fibers also affect the mechanical properties of the tissue 34. These structural features are not static but are constantly reshaped. As decidualization progresses, decidual fibroblasts secrete type IV collagen and laminin, forming a transient basement membrane-like structure 35, 36. The fibrous network becomes more extensible and less densely arranged, while matrix stiffness remains unchanged 37. It is worth noting that similar changes also occur in cervical tissue. Before delivery, the collagen arrangement becomes looser, the water content increases, and the content of sulfated glycosaminoglycans rises 38-40. These observations indicate that the remodeling of the uterine ECM may be a common biomechanical adaptation strategy during pregnancy.

**Figure 2:**
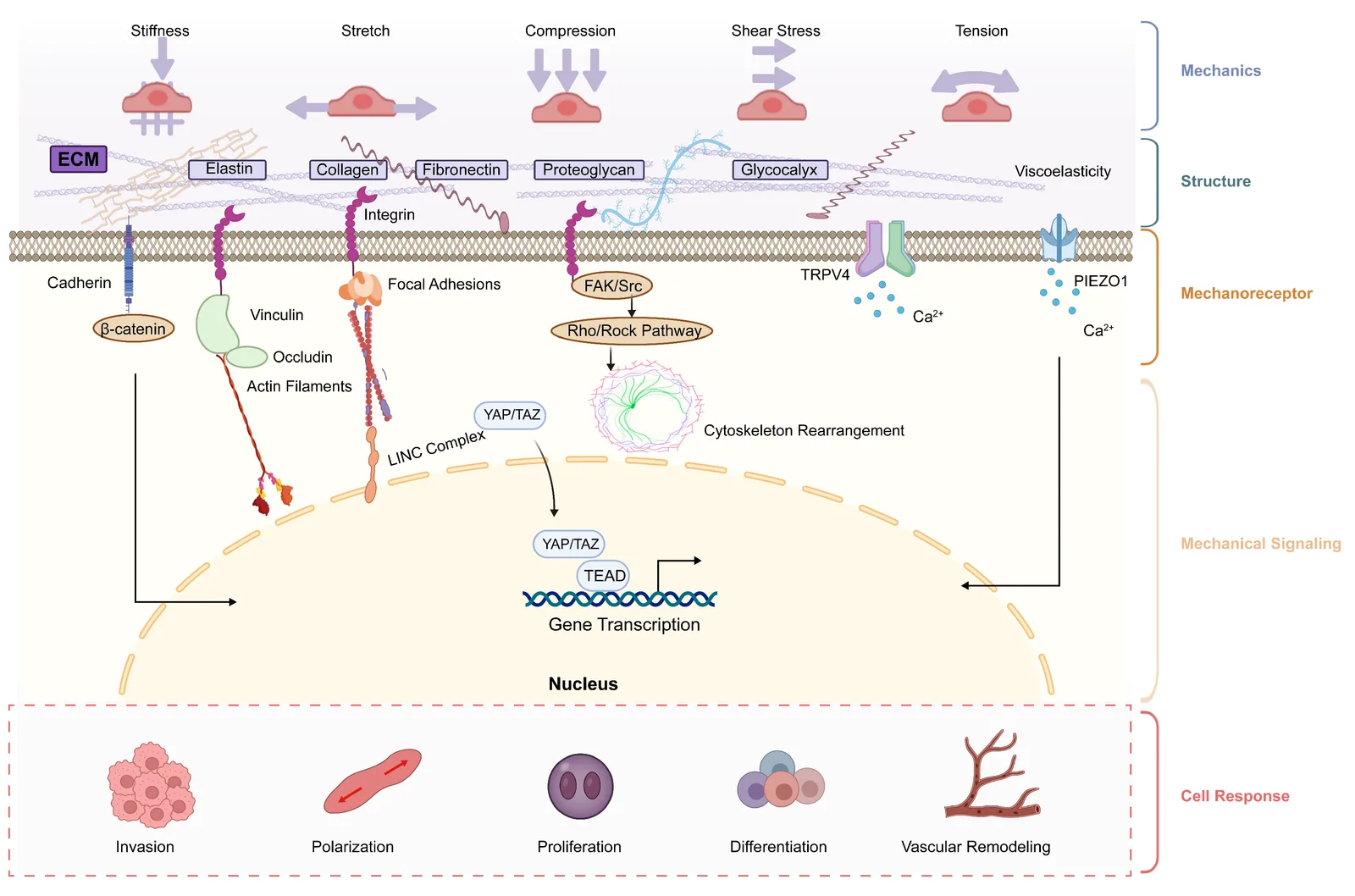
** The mechanics-structure-mechanoreceptor-mechano-signaling-cell response axis at the maternal-fetal interface.** This axis elucidates the relationship between the biomechanical properties of the maternal-fetal interface microenvironment and cellular function. Mechanical forces (extracellular matrix stiffness, compression, tensile force, tension, and shear stress) act on each cell at the maternal-fetal interface, triggering a mechanotransduction cascade. Alterations in extracellular matrix remodeling are sensed by mechanosensitive ion channels on the cell membrane (e.g., Piezo1, TRPV4), integrin receptors, and cell adhesion molecules, which transduce mechanical signals via calcium ion influx or conformational changes, activating cytoskeletal rearrangement and downstream pathways such as YAP/TAZ, leading to changes in gene transcription. Ultimately, these changes result in altered cell behaviors, including trophoblast invasion and fusion, morphological changes in stromal cells, shifts in immune cell polarization, and participation in spiral artery remodeling.

Mechanical cues within the uterine microenvironment are transmitted to cells through interactions with the ECM. Cells sense mechanical signals through mechanosensitive ion channels or integrin-based adhesion complexes. Among them, Piezo1 and Piezo2 are well-studied mechanosensitive ion channels 41. Experimental studies have shown that Piezo1 is mainly expressed in uterine smooth muscle cells and microvascular endothelial cells, and its expression is upregulated during term labor and downregulated during preterm birth 42. In contrast, Piezo2 is expressed in sensory neurons of the lower reproductive tract. Moreover, impairment of channel activity may be related to reduced contractility and altered labor progression 43. Mechanosensitive ion channels mediate the remote activation effect of stretch, thereby enabling synchronized contraction of the entire uterus 44. Integrins activate downstream signaling cascades, including focal adhesion kinase (FAK), which, in turn, regulates cytoskeletal organization and cell fate 45, 46. The intracellular mechanical transduction pathways, especially the Hippo signaling pathway and its effectors YAP/TAZ, act as a downstream pathway. They are the mediators through which cells respond to mechanical stimuli. Their activity is regulated by cytoskeletal tension and can be transferred to the nucleus upon activation, driving transcriptional programs related to cell proliferation and differentiation 47. *In vitro* studies have shown that ECM stiffness may affect the proliferation of endometrial stromal cells via the YAP signaling axis and promote uterine repair 48. These mechanical transduction mechanisms jointly ensure the foundation for uterine function during pregnancy** (Figure 2 and Table 2)**.

## Biomechanics Across Pregnancy: From Implantation to Parturition

### Embryo Implantation: Mechanical Dialogue Between Embryo and Endometrium

Embryo implantation is a dynamic mechanical interaction process involving forces generated by the embryo and physical constraints imposed by the uterus. Before implantation, the uterine contractions and ciliary movements propel the embryo through the reproductive tract. During the early stages of mammalian embryo development, the preimplantation embryo is encapsulated by the zona pellucida, which prevents it from directly sensing matrix stiffness or shear stress through integrins. *In vitro* models have shown that the zona pellucida in mouse stiffens after fertilization 49. And the mechanical properties of the zona pellucida may affect the development and positioning of early mouse embryos 50. As the embryo undergoes division and enters the maturation stage, this process is mainly driven by actin-myosin-dependent pulsed contractions. During this process, CDH1 regulates contractile force at cell-cell contact sites 51. Subsequently, the embryo undergoes asymmetric division, producing blastomeres with distinct contractile capabilities, thereby separating the fates of the inner cell mass (ICM) and the trophoblast cells (TE). In TE, YAP localization can enhance its mechanical sensing 52. During cleavage and differentiation into the blastocyst, the increase in intracavitary pressure promotes the formation of tight junctions through tension-cytoskeletal adhesion protein positive feedback loop 53. However, pressure above the threshold may lead to TE rupture, which might represent a protective mechanism. Periodic mechanical oscillations within the mouse blastocyst cavity could shape the spatial structure of the ICM lineage through fluctuations in cell-cell contact, cell movement, and PDGFRα/YAP signaling 54. In parallel, blastocyst expansion generates hydrostatic pressure, which may serve as a potential mechanical signal contributing to endometrial receptivity 55. The embryo is sensitive to the mechanical properties of the surrounding microenvironment. Three-dimensional *in vitro* studies have shown that culturing mouse embryos on a soft hydrogel mimicking uterine stiffness significantly increases blastocyst formation rate and hatching efficiency, while also increasing the number of trophoblasts 56. While, a stiffer matrix tends to inhibit development or promote osteogenic differentiation 57. This difference may be due to the embryonic stage. Mouse ESCs only acquire mechanical responsiveness after exiting the ground-state pluripotent state 58. Moreover, experimental studies using human pluripotent stem cells have shown that the soft matrix may regulate SMAD2/3 by upregulating the lncRNA LINC00458 to contribute to embryo development 59. Emerging research suggests that stress relaxation rate may be a more crucial parameter regulating implantation 60. When comparing across species, imaging platforms have also revealed specific differences in implantation modes: mouse embryos tend to exhibit expansive growth, whereas human embryos display more directional extension and adopt an embedded morphology 61. These observations highlight the diversity of implantation strategies across species and suggest that mechanical interactions between the embryo and uterine environment may vary accordingly.

At the maternal level, the endometrium undergoes significant structural and mechanical remodeling during the implantation window. It has been reported that ECM composition changes, including alterations in collagen subtypes in the endometrium during implantation and enrichment of type IV collagen in the embryonic basement membrane 62. The ECM can promote physical interactions between the embryo and the endometrium, influencing spatial positioning. Decidual stromal cells (dS) exhibit dual characteristics. On the one hand, they undergo matrix degradation to accommodate trophoblast invasion while maintaining an anti-matrix state to prevent excessive invasion 63. At the epithelial level, endometrial barrier properties are dynamically regulated. Redistribution of adherens junctions and desmosomes in endometrial epithelial cells shifts from apical to lateral, potentially reducing intercellular adhesion strength and facilitating trophoblast invasion 64, 65.

**Figure 3:**
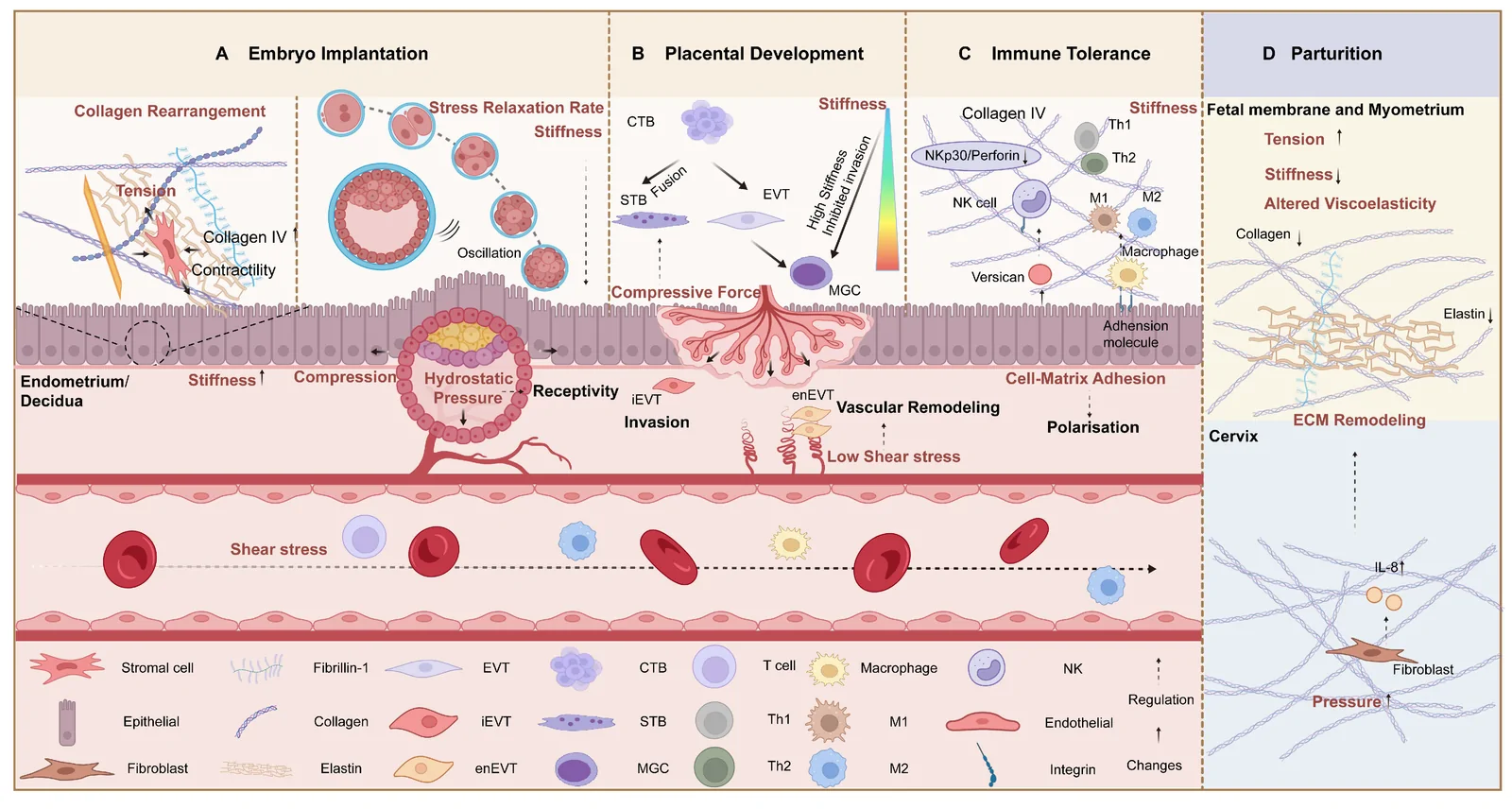
** Mechanical changes and mechanisms during normal pregnancy.** This figure illustrates the key mechanical events and potential mechanisms during the process from embryo implantation to delivery. A. The endometrium undergoes structural and mechanical remodeling. The collagen subtypes in the extracellular matrix change, and decidual stromal cells combine with this changed ECM, resulting in morphological changes. Before embryo implantation, the periodic mechanical oscillations of the blastocyst form the inner cell mass. The intracavitary hydrostatic pressure of the blastocyst increases, serving as a mechanical signal that promotes endometrial receptivity. B. Shear stress, stiffness gradients, and villous compressive forces regulate trophoblast differentiation, migration, and vascular remodeling processes. C. The stiffness and composition of the extracellular matrix regulate the polarization and cytokine secretion of immune cells, such as macrophages and NK cells, through adhesion molecules. D. The tension exerted on the myometrium and fetal membranes, along with their intrinsic stiffness and viscoelasticity, as well as the mechanical compression on the cervix, participate in the process of parturition by modulating extracellular matrix remodeling through mechano-immunological mechanisms.

Embryonic and maternal cells jointly regulate and respond to the mechanical microenvironment at the maternal-fetal interface. During trophoblast invasion, dynamic changes in type IV collagen and fibronectin-1, as well as vascular remodeling, have been reported. These changes may be related to the regional differences in human decidual stiffness (basal decidua *vs.* parietal decidua: 1250 Pa *vs.* 171 Pa) 66. Trophoblasts exhibit elongated pseudopodia and increased physical stiffness to reduce fluid shear-induced damage and enhance interaction with the surrounding matrix. In parallel, endometrial epithelial cells undergo changes in non-polarized actin cytoskeletal organization, apically localized integrins, and calcium signaling, which may affect tight junctions at the maternal-fetal interface 67, 68. Microfluidic models further demonstrate that shear stress and trace forces may contribute to decidualization by regulating cytoskeletal rearrangement 69. Endometrial epithelial cells express PIEZO1 and epithelial sodium channels (ENaC), converting mechanical stimuli into biochemical signals, including calcium ion influx, to regulate prostaglandin E2 production during early implantation 65, 70. While deletion of Piezo1 in mice may even result in embryonic death. After implantation, the mechanical constraints imposed by the maternal uterus may affect the formation of the body axis in early mouse embryos by limiting developmental space 71. Most of these findings come from animal models or *in vitro* systems, while human data are relatively sparse. Nevertheless, the overall situation indicates that ECM properties and intercellular connections affect the balance of local forces and regulate decidualization and blastocyst implantation **(Figure 3A)**.

## Placental Development: Forces in Trophoblast Invasion and Vascular Remodeling

The placenta is a critical organ during pregnancy, exhibiting compressive symmetry, nonlinear viscoelasticity, and strain-rate dependence 72, 73. These properties ensure the structural integrity under physiological loads. During placental development, cytotrophoblast (CTB) gradually fuses into syncytiotrophoblast (STB) or differentiates into extravillous trophoblast (EVT), and EVT further differentiates into endovascular (enEVT) and interstitial (iEVT) subtypes. These cells invade the decidua and maternal vasculature, remodeling spiral arteries to establish an efficient maternal-fetal circulation 11, 74. During this process, trophoblasts appear to sense and respond to mechanical signals in the surrounding microenvironment. Cell surface integrins and intracellular focal adhesion complexes play a role and exhibit lineage-specific expression patterns. For example, VCT preferentially expresses α4β6, whereas EVT expresses α5β1, which is associated with the invasive phenotype 75. In addition, ECM stiffness has been reported to modulate the morphology and invasion ability of trophoblasts. A certain high stiffness can promote the directional behavior of EVT 76. While upon encountering regions of higher stiffness, such as the uterine myometrium, EVT may exhibit inhibited migration and fuse into multinucleated giant cells. Similarly, matrix stiffness can regulate the fusion and morphological transformation to STB form and hormone release through myosin-II 77. STB simultaneously expresses mechanosensitive ion channels (Piezo 1, Polycystin 2, TRPV6) and motility proteins related to primary cilia (Dynein 1, IFT88, Kinesin 2) 78, 79. These molecules may be crucial for the key mechanical transduction. Moreover, experimental and computational studies have shown that directional compressive forces could enhance trophoblast fusion efficiency and villous formation by syndecan1 and E-cadherin *in vitro* 80. This indicates a close connection between multiple mechanical signals and the behaviors of different trophoblast types.

Hemodynamic factors provide another perspective on mechanical regulation during placental development. During pregnancy, the uterine spiral arteries undergo significant remodeling. Incomplete vascular circumferential adaptation in mice, potentially alters stress distribution within the uteroplacental circulation 81. In human, structural changes, including the replacement of vascular smooth muscle with fibrinoid material and the formation of the Nitabuch layer, are associated with increased local tissue stiffness 82, 83. Shear stress, arising from increased maternal blood flow, modulates cellular functions through distinct mechanisms in a cell-type-specific manner. In endothelial cells, Piezo1 mediates shear stress-induced calcium influx, thereby promoting nitric oxide production and vascular adaptation in rats 84. The same holds true in human placental vascular endothelial cells, and may be associated with small for gestational age babies 85, 86. Pig models suggested that mechanical forces generated by increased blood flow at the uteroplacental interface may trigger focal adhesion assembly and actin polymerization, thereby forming sculp folds 87. A computational model suggested that the optimal morphology of human terminal villi is also regulated by the pressure gradient and changes in blood flow velocity, and driven by shear forces and deformation 88. In the intervillous space, shear stress upregulates angiogenic factors such as PlGF via the cAMP-PKA pathway, thereby participating in the differentiation and functional maturation of human STB 7, 89. Additionally, shear stress reprograms STB metabolism by inhibiting glycolysis and activating GOT-mediated amino acid metabolism, an effect dysregulated in fetal growth restriction (FGR) 90. enEVT may respond to low shear stress by inhibiting directed migration while enhancing endothelium-derived chemotactic gradients during vascular remodeling 11, 91. In contrast, elevated shear stress may act as a biomechanical signal to terminate spiral artery remodeling 6. These mechanical-biological feedback mechanisms ensure the effective establishment and development of the placental structure and maternal-fetal circulation **(Figure 3B)**.

## Pregnancy Maintenance: Biomechanic-Immune Crosstalk

Immune tolerance at the maternal-fetal interface is essential for maintaining pregnancy, allowing the semi-allogeneic fetus to coexist with the maternal immune system. This process involves decidual natural killer (dNK) cells, macrophages, and T cells, forming an immunosuppressive microenvironment 92. Emerging evidence suggests that immune cells are sensitive to mechanical cues, including stiffness, shear stress, spatial confinement, and tension. Insights from other fields, such as tumor immunology and computational modeling, suggest that these factors influence immune cell migration, activation, and polarization through mechanotransduction pathways 93. However, their relevance to pregnancy requires further investigation. At the maternal-fetal interface, immune cells are embedded within a dynamically remodeling ECM, which may provide both structural support and mechanical signals. Decidual macrophages predominantly exhibit an M2-like anti-inflammatory phenotype, which has been associated with placental development and immune regulation during early pregnancy 94. *Ex vivo* studies suggest that matrix physical properties may influence macrophage polarization and cytokine secretion by the Piezo1-YAP axis 95, 96. A pregnancy-related study further indicates that decidual stromal cells may influence CD16+ decidual macrophages through ECM-adhesion molecule interactions, promoting an immunoregulatory phenotype during early pregnancy 97. ECM components may also regulate immune cell function. Collagen has been reported to reduce dNK cytotoxicity by downregulating molecules such as NKp30 and perforin, while influencing cytokine production, such as IFN-γ and TNF-α 98. Proteoglycans such as versican, secreted by endometrial stromal cells, have been implicated in regulating the proliferation of tissue-resident NK cells and spiral artery remodeling 99. The ECM and its physical properties seem to be a key regulator hub for immune tolerance in pregnancy. Different responses of distinct immune cells to the same mechanical signal enable fine-tuned regulation of immune homeostasis **(Figure 3C)**.

## Parturition: Mechanical Remodeling of Cervix and Fetal Membranes

The cervix, the fetal membranes, and the uterus form a complete mechanical coupling system. This system can maintain the integrity of pregnancy and coordinate the delivery process. The cervix provides structural support, the fetal membranes help to bear the load, and the uterus generates contraction force.

The fetal membrane is a thin layer of tissue composed of the inner amnion and the outer chorion. There is a movable interface between the two, allowing stress distribution during pregnancy. Among them, the amnion is the main load-bearing layer, and the chorion serves as the buffering layer 88. The structural integrity of both depends on their tensile strength, extensibility, and viscoelasticity, which are jointly regulated by the contents of elastin and collagen 100, 101. As amniotic fluid volume increases and the fetus grows, the fetal membranes must resist the pressure within the uterus and adapt to the gradually increasing mechanical stretching. *In vitro* experiments have shown that before labor, the intermediate layer of the amnion undergoes collagen degradation and softening, with a significant reduction in fiber density, resulting in decreased stiffness, which is the basis for labor initiation 101. At full term, the rupture of the amnion is related to the physiological weakening of its mechanical properties.

Similarly, during normal pregnancy, the cervix remains closed and firm, and its mechanical strength is mainly maintained by type I and type III collagen 102. During late pregnancy and delivery, the cervix undergoes adaptive remodeling. It goes through the stages of softening, maturation, dilation, and postpartum repair, characterized by decreased tissue stiffness, increased viscoelasticity, increased compliance, and decreased load-bearing capacity 103, 104. This behavior is driven by time-dependent matrix reorganization 105, 106. Collagen transforms from a highly ordered to a more disordered network, with increased fiber dispersion and solubility. At the same time, EP4 regulates the stretching strength of the rat cervix to adapt to the stress required for delivery by modulating collagen rearrangement and microstructural changes 107. At the molecular level, it is driven by increased matrix metalloproteinase activity and decreased tissue inhibitor expression 108, 109. The expression of proteoglycans and collagen fibers in the rat cervix increases during the delivery period, and their interaction promotes cervical dilation, rapidly decreasing to the non-pregnant level one day after delivery 110. Cervical shortening is most significant in the later stages of pregnancy, reflecting the cumulative effects of mechanical load and tissue remodeling 111. Mechanical force participates in cervical remodeling through feedback mechanisms. The gradual increase in uterine volume and intrauterine pressure increases the pressure on the cervix, affecting the coupling of uterine contractions and cervical dilation 112, 113. Mouse models indicate that the cervical tissue is anisotropic, and its axial contraction force and circumferential stiffness play a key role in maintaining homeostasis 114. An increase in intrauterine pressure and mechanical stimulation of the cervix by the fetal head can activate mechanosensitive pathways in cervical fibroblasts, regulate IL-8 release, and thereby modulate ECM synthesis and degradation 115. This mechanical-biochemical feedback loop coordinates uterine contractions, fetal membrane tension, and cervical remodeling during delivery. Before full-term delivery, there is a clear "weak zone" above the cervix in the area of the fetal membranes, which is the starting point of physiological rupture 116. The cervix exhibits regional-specific structural and functional differences. The upper part may participate in the regulation of labor through contraction activities 117. The three-dimensional finite element model in the late pregnancy further confirms that intrauterine pressure acts on both the fetal membranes and the cervix, and cervical shortening reversely affects the stress distribution in the weak zone of the fetal membranes, suggesting that studying a single tissue in isolation cannot reflect the true interactive nature of the prenatal mechanical environment 108.

Mechanical forces have been suggested to interact with electrophysiological pathways in regulating uterine contraction. For example, multiscale modeling studies have indicated that tensile forces may contribute to synchronized whole-uterine contraction 44. Clinical findings have indirectly validated this theory, showing that mechanical stimulation (e.g., abdominal pressure) can directly induce bioelectrical activity, confirming the mechanosensitivity of the uterus 118. These findings suggest the presence of a potential feedback system in which mechanical forces, cellular responses, and electrophysiological signals are dynamically integrated **(Figure 3D)**.

## Biomechanical Dysregulation in Pregnancy Complications: From Adaptation to Maladaptation

### Implantation Failure

**Figure 4:**
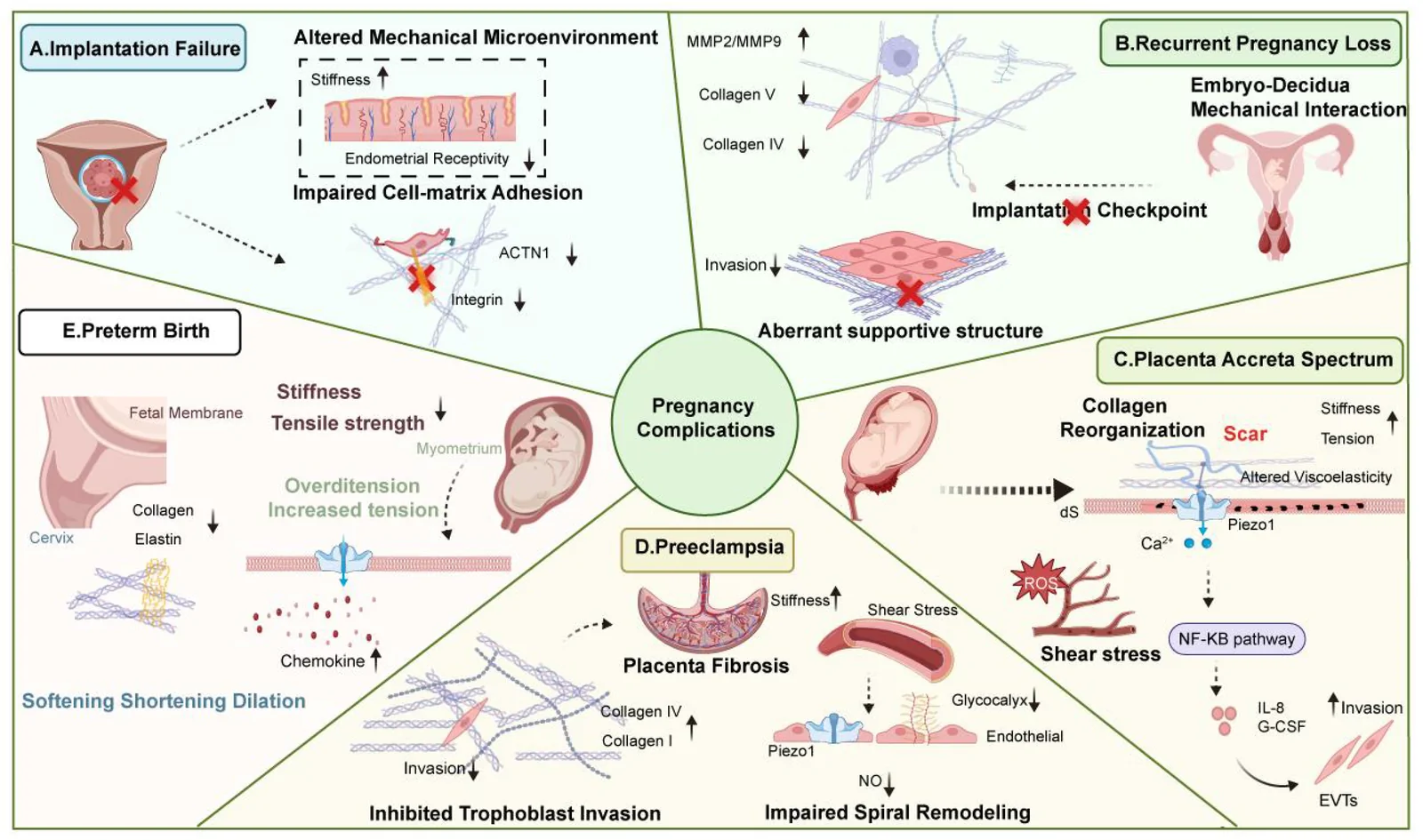
** Mechanical mechanisms underlying pregnancy complications.** This figure summarizes the mechanisms associated with several pregnancy complications related to biomechanical abnormalities. A. Alterations in the mechanical microenvironment and impaired cell-matrix interactions are associated with implantation failure; B. Abnormal extracellular matrix remodeling and aberrant supportive structures disrupt the mechanical homeostasis of the maternal-fetal interface, contributing to recurrent pregnancy loss; C. Altered mechanical properties of scar tissue, manifested as increased stiffness, reduced tension, and disorganized collagen architecture, activate Piezo1 ion channels, leading to the release of pro-inflammatory factors, regulating abnormal trophoblast invasion, and contributing to placenta accreta spectrum (PAS); D. Shear stress resulting from abnormal vascular remodeling and increased stiffness from aberrant extracellular matrix remodeling inhibit trophoblast invasion and spiral artery remodeling, contributing to preeclampsia (PE); E. Preterm birth is associated with weakened mechanical properties of the fetal membranes, cervical remodeling, and uterine contractions induced by abnormal tension.

Embryo implantation requires a coordinated interaction between the embryo and the endometrium. Changes in uterine mechanical activity, including uterine contractility and endometrial stiffness, may be associated with implantation outcome. Fanchin 119 and Chung's team 120 suggested that a high-frequency contraction pattern may lead to implantation failure. In contrast, Blank 121 proposed that high-frequency, low-amplitude uterine activity is beneficial for implantation. The inconsistency may be due to differences in observation time and measurement methods. In addition, mechanical stimulation of the endometrium has been suggested to improve endometrial receptivity to reduce implantation failure 122. At the cellular level, cell-matrix interactions play a critical role in mediating embryo attachment. Studies in mouse models have revealed that preimplantation embryo movement comprises three stages: the entry phase, the unidirectional clustering movement phase, and the bidirectional dispersal phase. Among these, embryo clustering movement depends on myometrial contractions, whereas dispersal relies on the LPAR3 signaling pathway. While disruption of integrin-mediated force transmission impairs implantation in mouse models 123. In RIF patients, it has been further found that upregulation of anti-adhesion proteins PDX and α-actinin-1 impairs endometrial epithelial cell adhesion 124, 125. Collectively, these findings suggest that RIF may be associated with alterations in the mechanical microenvironment and impaired cell-matrix interactions, although the causal relationships remain to be fully established **(Figure 4A and Table 2)**.

### Recurrent Pregnancy Loss

Following successful implantation, maintenance of early pregnancy requires coordinated interactions between the embryo and the maternal decidua. If this interaction is abnormal, it will lead to recurrent pregnancy loss (RPL). RPL is a multifactorial condition, with a substantial proportion of cases remaining unclear. Congenital and acquired uterine morphology abnormalities are associated with RPL. Changes in morphology may affect the uneven distribution of tension within the uterine cavity, influencing uterine cavity mechanics and, in turn, embryo implantation and stability 126. Emerging evidence suggests that alterations in ECM composition may be associated with RPL. Reduced expression of type IV and type V collagen has been observed in the decidua of RPL patients, indicating potential changes in tissue structure and mechanical properties 127-129. While upregulation of MMP-9 in RPL suggests that an imbalance between ECM degradation and synthesis may weaken the ECM and interfere with the embedding processes 130. In the RPL mouse, supplementation with recombinant human collagen can regulate the Th17/Treg balance, reduce the incidence of retained fetuses, reshape the immune microenvironment at the maternal-fetal interface, and improve reproductive capacity 131. The endometrium functions as a selective interface that integrates biochemical and mechanical signals during implantation. It has been proposed that this “implantation checkpoint” may involve not only molecular signaling but also mechanical interactions between the embryo and the endometrium 132. While alterations in tissue structure and cell-matrix interactions may contribute to RPL, direct evidence linking specific mechanical parameters to RPL remains limited. We hypothesize that the mechanism of selective elimination of low-quality embryos may also involve abnormal mechanical interactions between the embryo and the endometrium **(Figure 4B and Table 2)**.

### Placenta Accreta Spectrum (PAS)

Normal placental development requires tightly regulated trophoblast invasion and coordinated remodeling of the maternal vasculature. Under physiological conditions, EVT invade the decidua and remodel spiral arteries to establish a low-resistance, high-capacity circulation.

Cesarean Scar pregnancy (CSP) and placental accreta spectrum (PAS) are associated with abnormal implantation and excessive trophoblast invasion 133. Scar has been identified as a common risk factor for both conditions 134. *In vitro* and *in vivo* experiments have demonstrated that scars exhibit altered mechanical properties, including increased stiffness and changes in collagen organization 135-137. While no impact of CS scars on uterine motility patterns has been identified, regional anterior-posterior wall motion asymmetry and abnormal viscoelasticity of the lower uterine segment muscle exist, potentially leading to subsequent abnormal embryo implantation 138, 139. These changes may influence the local microenvironment at the implantation site. Alterations in tissue mechanics may affect trophoblast behavior. Experimental observations suggest that there may be differences in the invasive capacity of trophoblast cells between scar and non-scar tissue. Compared with non-scar regions, the invasive ability of trophoblasts in scar tissue is enhanced, and restoring normal tissue structure appears to reverse this behavior 140. *In vitro* scar models have shown that Piezo1 participates in trophoblast cell responses to mechanical stimuli by secreting IL-8 and G-CSF 8. In addition to local tissue properties, abnormal vascular remodeling contributes to altered hemodynamic conditions in PAS. Impaired spiral artery remodeling may result in increased blood flow velocity and altered shear stress within the intervillous space 141. On one hand, reactive oxygen species (ROS) are generated, disrupting ECM structure and altering EVT invasion signals 142. On the other hand, increased shear stress and mechanical injury from unreconstructed arteries induce lacunar formation and thrombosis. These hemodynamic disturbances may impair placental function and are associated with an increased risk of adverse pregnancy outcomes 143.

Collectively, PAS may involve complex interactions between altered ECM structure, abnormal tissue mechanics, and dysregulated hemodynamic forces. These findings support a conceptual framework in which the local microenvironment (“soil”), trophoblasts (“seeds”), vascular flow (“flow”), and ECM structure (“scaffold”) interact to shape placental implantation. Within this context, a mechanical-biological imbalance of the endometrium may influence trophoblast behavior, contributing to disease. Therefore, in clinical practice, we need to assess the mechanical state of the uterine scar to determine the risks of PAS or uterine rupture **(Figure 4C and Table 2)**.

### Preeclampsia

Preeclampsia (PE) is associated with impaired placental vascular remodeling and alterations in the mechanical properties of the uteroplacental circulation. Clinical and experimental studies have reported reduced uterine artery dilation, increased vascular stiffness, and altered pulse wave transmission in early PE pregnancies 81, 144, 145. In addition, abnormal shear stress patterns have been associated with PE and fetal growth restriction, suggesting that hemodynamic forces may be altered in this condition 146. At the mechanistic level, endothelial cells are known to respond to altered mechanical cues. Piezo1 has been implicated in shear stress-induced calcium signaling and nitric oxide (NO) production via the Ca^2+^/eNOS pathway, which is involved in vascular function. Rat models suggest that disruption of the Piezo1-NO axis may be associated with impaired vasodilation in PE 9, 147. Other mechanosensitive channels, such as TRPV4, have also been reported to participate in NO-mediated vascular responses, and their altered activity has been associated with early-onset PE 148. Furthermore, the structural components of the vascular microenvironment may also affect mechanical signal transmission. In PE, an abnormal glycocalyx may impair endothelial cells' ability to sense shear stress and regulate NO production 149. Additionally, ECM remodeling and fibrosis were observed in PE placentas, manifested as increased collagen deposition and tissue stiffness. Increased COL4A1 and COL4A2 expression has been associated with impaired vascular remodeling 150, 151. *In vitro* studies suggest that hypoxia may promote ECM deposition via TGF-β1 signaling, contributing to placental fibrosis 152, 153. The mechanically sensitive signaling pathway can also regulate cellular metabolism. Recent studies have shown that the Piezo1/YAP/PFKFB3 axis may regulate the glycolysis of endothelial progenitor cells (EPCs) and promote the formation of new blood vessels in the placenta during PE 154. However, these findings are primarily derived from experimental systems and require further validation in humans. Beyond the uteroplacental circulation, mechanical alterations were also observed in fetal tissues. Increased collagen fiber density and stiffness in the umbilical artery, along with reduced Wharton's jelly area and elasticity, have been observed in PE, which may have implications for fetal cardiovascular development 155. The abnormality in the above-mentioned behavior may interfere with normal placental development, leading to abnormal mechanical properties. This also explains why PE presents abnormal mechanical properties of the placenta 88. The abnormality of the placenta, as a bridge for transporting nutrients and connecting the mother and the fetus, reflects the mechanism of abnormal fetal outcomes. For example, the stiffness and viscosity of the placenta in cases of fetal growth restriction decrease 156, 157.

Collectively, these findings suggest that PE may involve interactions between altered ECM remodeling, abnormal vascular mechanical properties, and dysregulated mechanosensory pathways. However, whether these mechanical alterations represent primary drivers of disease or secondary responses remains unclear. Therefore, mechanical parameters are currently better considered as potential contributors or biomarkers **(Figure 4D and Table 2)**.

### Preterm Premature Rupture of Membranes and Preterm Birth

Before delivery, a series of coordinated mechanical and biochemical changes occur, including fetal membrane relaxation, cervix remodeling, and uterine contractions. These processes are tightly regulated to ensure term delivery. Preterm birth (PTB) is associated with conditions involving increased mechanical load, such as multiple pregnancies and polyhydramnios, suggesting that uterine overdistension may alter the mechanical environment of pregnancy 158. One cause of PTB is preterm premature rupture of membranes (PPROM). The high tensile stiffness and rupture critical tension of the fetal membranes depend on the content and cross-linking structure of the mature collagen fiber network. When these two are abnormal, the tensile strength and stiffness of the fetal membranes decrease, causing physiological pressure-induced membrane rupture 159. In the tissues obtained from vaginal delivery with spontaneous rupture of the fetal membranes, COL1A1 and COL1A2 expression were significantly lower than those from elective cesarean section without fetal membrane rupture 160. Computational biomechanical modeling has further revealed that lubrication at the chorion-amnion interface decreases, leading to chorion thinning and potentially increasing fetal membrane stress and rupture 161. This suggests the importance of tissue structure and mechanical properties in maintaining the integrity of the fetal membranes. The amnion has a certain degree of toughness, and a macroscopic mechanical defect of the amnion alone cannot cause rupture 162. Therefore, PPROM is more likely to result from a biochemical imbalance accompanied by increased mechanical load. Another cause of PTB is cervical factors. Alterations in the collagen and elastin networks of fetal membranes may also lead to premature softening, shortening, and dilation of the cervix 101. A clinical trial found that the collagen concentration in the cervix of women with cervical insufficiency (CI) was lower, and the tissue strength was reduced 163. Perhaps the abnormal mechanical properties triggered premature contractions of the uterus, resulting in miscarriage and PTB. In addition, the uterine myometrium is not a passive load-bearing structure that bears tension; rather, it actively senses and responds to mechanical stretch 164. At the cellular level, mechanical stretch has been reported to regulate chemokine expression and inflammatory signaling pathways, which may contribute to the initiation of uterine contractions 165, 166. In addition, stretch-induced electromechanical coupling may activate calcium channels in uterine smooth muscle cells, contributing to contractile activity 37. Experimental studies suggest that Piezo1 activation modulates uterine contractility and inflammatory signaling 154, 167. Preterm birth is likely the result of immune activation under mechanical load **(Figure 4E and Table 2)**.

## Translational Research and Future Directions

### Biomechanical Monitoring

**Figure 5:**
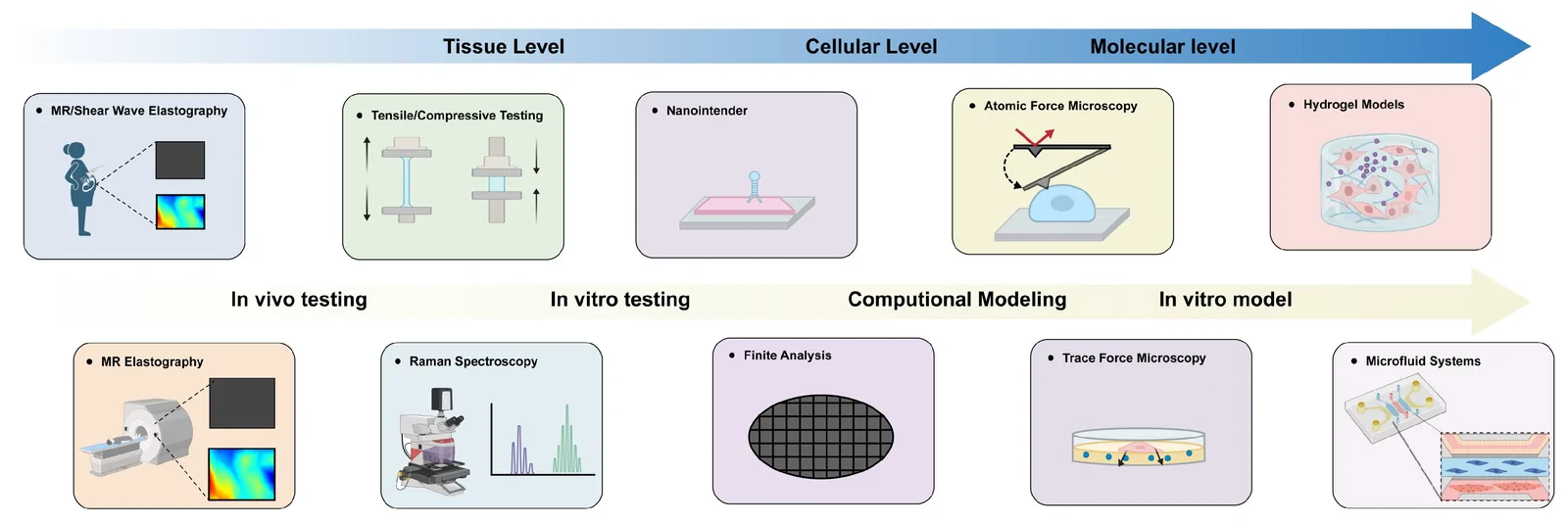
** Multi-scale biomechanical tools for mechanical properties.** This figure provides an overview of the tools that can be applied to monitor the mechanical properties of the maternal-fetal interface and explore mechanical mechanisms at the tissue level, cell level, and molecular level. It covers macroscopic elastic imaging, microscopic atomic force microscopy, and hydrogels and microfluidic models for dynamic regulation of mechanical properties.

Currently, certain biophysical imaging techniques have enabled non-invasive assessment of tissue mechanical properties during pregnancy **(Figure 5)**, but their clinical maturity varies considerably. Strain and shear wave elastography have been applied in several preliminary clinical exploratory studies to evaluate cervical softening and predict preterm birth risk 168-170. *Ex vivo* experiments have further characterized the spatial variation in cervical shear wave velocity and proposed it as a potential quantitative indicator of cervical softening 171. In addition, ultrasound imaging has also been used to detect changes in stiffness at cesarean section scars 172. *In vivo* measurements of cervical stiffness using ultrasound-based maximal deformation and the aspiration method have also been used to predict preterm birth, and they may perform better than elastography 173. However, most of these applications are currently based on small sample studies. Raman spectroscopy has been shown to capture changes in collagen structure, water content, and lipid signals in the pregnant mouse cervix, and these changes correlate with decreases in cervical stiffness during pregnancy 174. An MRI-based parametric solid model can reconstruct a three-dimensional uterine structure during late pregnancy to simulate tissue deformation and mechanical heterogeneity. Still, some deviation between the models and the actual situation remains 175. A dual-arm nanorobot system enables non-invasive measurement of embryo mechanics, but it is still at an early experimental stage 176. Tensile tests and nanoindentation mainly measure the material properties of each layer of the uterus *in vitro* 28. The future applications of these technologies may enable *in vivo* dynamic mechanical monitoring of the uterus during pregnancy. Moreover, they can provide key biomechanical indicators to determine the optimal time of delivery and prevent complications (e.g., cervical stiffness and placental elasticity). Given the differences in mechanical properties between *in vivo* and *ex vivo* conditions, future work should focus on standardizing operating protocols, establishing quantitative thresholds, and basing them on human samples to explore the feasibility of mechanical monitoring.

### Experimental Models and Mechanobiology Tools

At the microscale, some experimental tools have helped us explore microscopic mechanics **(Figure 5)**. Atomic force microscopy (AFM) and traction force microscopy are measurement tools; the former directly measures local stiffness and adhesion forces of individual cells or the ECM, while the latter can visualize the forces exerted by cells during migration or contraction on the substrate 61, 177. Additionally, a microfluidic system can simulate cells under dynamic mechanical conditions, such as shear stress and concentration gradients, to explore changes in vascular remodeling during pregnancy 178. The combination of organoid and hydrogel models enables the adjustment of parameters, such as stiffness and viscoelasticity, to simulate the regulatory role of cell behavior under various mechanical conditions 60, 179. Currently, these methods are mainly used to explore the mechanism of the mechanical signal axis. Nevertheless, *in vitro* models cannot fully capture the spatiotemporal complexity *in vivo*, making it difficult to recapitulate physiological phenomena and limiting the extrapolation of research results to humans.

### Therapeutic Strategies Targeting Mechanics

Some scholars have begun to attach importance to biomechanics in clinical practice during pregnancy 180. Physical biomechanical interventions have already been preliminarily applied clinically. For example, the silicone double-balloon catheter promotes cervical ripening through mechanical cervical dilation 181. In contrast, drugs targeting mechanotransduction signaling pathways remain largely at the concept stage. The Piezo1 agonist Yoda1 can restore mechanosensitivity of Piezo1 mutants, suggesting its potential therapeutic effect on hydrops fetalis 182. Vedolizumab targets integrin α4β7 and blocks MAdCAM-1-mediated cell adhesion and invasion 183. *In vitro* experiments have shown that it inhibits trophoblast invasion, but its safety *in vivo* has not been validated. In the future, perhaps targeted drugs targeting the ECM, or drugs targeting the core mechanical transduction elements, could be considered to intervene in the mechanical characteristics and in cells' perception and response to the mechanical environment. Physical interventions target the upstream source directly and act quickly. Molecular interventions focus on sensing and transducing mechanical signals. Of note, we have to carefully monitor the safety of applying mechanically targeted drugs during pregnancy.

### Modeling and Future Directions

Computational modeling offers a unique perspective for research in pregnancy biomechanics. The microstructural fiber network model of fetal membrane rupture shows that the high toughness of the amnion arises from non-affine deformation and crack deflection of its collagen fibers. The multiscale model of the maternal-fetal interface simulates the process by which trophoblasts gradually remodel blood vessels 184. However, current models remain partially dependent on parameter selection, which limits their physiological relevance and extrapolation capability. Future efforts should focus on constructing molecular-cellular-tissue system models that comprehensively reveal the biomechanical behavior of the uterus under physiological and pathological conditions.

## Conclusions

Overall, biomechanics is not merely a passive factor in pregnancy. On the contrary, it is involved in regulating key events throughout the entire pregnancy process. Currently, most evidence on the correlation between mechanics and pregnancy comes from animal models and *in vitro* experiments. Nevertheless, these studies have identified several possible mechanical mechanisms, including PIEZO1, integrins, and YAP/TAZ, which seem to play important roles in trophoblast cell invasion, vascular remodeling, immune tolerance, and cervical maturation. Moreover, abnormalities in stiffness, shear stress, and tension may be related to the occurrence of pregnancy complications. Future research should combine advanced biomechanical modeling with validation using real human samples to confirm the role of mechanical forces. Additionally, the feasibility of targeting mechanosensors such as PIEZO1 and YAP/TAZ for the treatment of pregnancy complications should be explored.

## Figures and Tables

**Table 1:** Representative Biomechanical Properties of the Uterus

Tissue Region	Mechanical Property^*^	Measurement Methods (Quantitative Metric)	Species	Findings
The entire uterine wall	Tensile Strength ^a^	*Ex vivo* Dynamic Uniaxial tension to failure (Peak true tensile stress, kPa; Elongation at break, dimensionless/%)	Human (Fresh tissue)	Pregnant lower uterine segment: Nonlinear stress-strain behavior ^b^; Peak true tensile stress: 656.3±483.9kPa; Elongation at break: 32.7%±11.2% (range: 14-45%) 25
	Stiffness ^c^ Viscoelasticity ^d^	*In vivo* and *Ex vivo* IFEA (Model-derived parameter, N/m^2^; Maximum elongation ratio, dimensionless)	Human	Non-pregnant uterus: Regional stiffness-related parameter: Fundus (3477 N/m^2^) > Dorsal region (3044 N/m^2^) > Ventral region (1239 N/m^2^); Apparent modulus decreased markedly *ex vivo*; Maximum elongation ratio ^e^: 1.1-1.3 *in vivo*, 1.2-1.45 *ex vivo* 18
Uterine Layers	Stiffness ^c^ Viscoelasticity ^d^	*Ex vivo* Microindentation and PVE model (Elastic modulus, Pa; Viscoelastic ratio, dimensionless)	Rhesus macaque (Frozen tissue)	Layer-specific stiffness: Endometrium / Decidua < Myometrium < Perimetrium, (range 10-10^4^ Pa); Viscoelastic Ratio: Endometrium/Decidua < Myometrium, Perimetrium (range 0.3-0.6); The endometrium and pregnant decidua are the least stiff, most viscous, least diffusible, and most hydrated layers; No significant gestational changes in elastic modulus, viscoelastic ratio, permeability, or diffusivity were observed 10
	Stiffness ^c^	*Ex vivo* AFM indentation (Apparent elastic modulus, Pa)	Human (Fresh tissue)	Region-dependent stiffness: Decidua Basalis (1250Pa) > Endometrium (250Pa) > Placenta (232Pa) > Decidua Parietalis (171Pa) 66
	Viscoelasticity ^d^ Anisotropy ^f^	*Ex vivo* Dynamic Uniaxial cyclic compression (Complex modulus, kPa)	Human (Fresh tissue)	Myometrium, non-pregnant uterus: Frequency-dependent Viscoelasticity; Complex modulus increased with frequency: 28 -35 kPa at 0.1 Hz and 78-95 kPa at 100 Hz; Parallel direction > Perpendicular direction 21
	Stiffness ^c^ Compliance ^g^ Tensile Strength ^a^	*Ex vivo* Uniaxial tensile and compression test (Elastic modulus, kPa/psi; Tensile strength, MPa/psi)	Human (Fresh tissue)	Myometrium: Stiffness: Non-pregnancy-965kPa (140psi) > Pregnancy-586kPa (85psi); Tensile strength: 0.55-2.07MPa (80-300psi); The outer layer is softer in compression, stiffer in tension 19
	Compliance ^g^ Stress Relaxation ^h^	*Ex vivo* Passive stretch test (Passive length-tension behavior; Stress relaxation rate, %)	Human (Fresh tissue)	Myometrium Pregnancy increased passive compliance, as shown by flatter passive length-tension curves 20, 22; Stress Relaxation Rate: Non-pregnancy (27.24%) > Pregnancy (22.67%)^27^
	Stiffness ^c^ Viscoelasticity^ d^	*In vivo* 3DMMRE (Magnitude of complex shear modulus, kPa; Phase angle, dimensionless)	Human	Myometrium, non-pregnant uterus: Magnitude of complex shear modulus: Uterine corpus (2.58±0.52kPa) > Cervix (2.00±0.34kPa); Magnitude of shear modulus: Endometrium-Proliferative phase (3.34±0.42kPa) > Secretory phase (1.97±0.34kPa); Myometrium-Proliferative phase (3.01±0.26kPa) > Secretory phase (2.22±0.26kPa); Phase angle: Myometrium (0.57±0.09) > Endometrium (0.32±0.10) 23
	Equilibrium stress ^I^ Anisotropy ^f^	*Ex vivo* Spherical indentation, Uniaxial tensile test, IFEA (Equilibrium stress, kPa)	Human (Fresh tissue)	Equilibrium stress: Non-pregnant (7.69±5.89kPa)> Pregnant (3.04±0.80kPa) 37
	Stiffness ^c^ Viscoelasticity ^d^	*Ex vivo* nanoindentation, PVE model (Elastic modulus, kPa; Viscoelastic ratio, dimensionless)	Human (Frozen tissue)	Elastic modulus: Endometrium (0.143±0.04kPa) < Decidua (0.352±0.07kPa) < Perimetrium (0.365±0.17kPa) < Non-pregnant myometrium (0.738±0.45kPa); Viscoelastic Ratio: Endometrium (0.430±0.02) < Decidua (0.485±0.03) 28

*Definition and units of mechanical parameters ^a^ Tensile strength, kPa/MPa/psi: The maximum tensile stress sustained by tissue before rupture; ^b^ Stress-strain behavior, stress in kPa and strain dimensionless or %: The relationship between applied stress and tissue deformation during loading; nonlinear stress-strain behavior indicates that tissue stiffness changes with increasing deformation; ^c^ Stiffness-related metrics, N/m^2^ or Pa/kPa/MPa/psi: The resistance of tissue to deformation. In this table, stiffness-related behavior is reported according to the original studies as model-derived stiffness-related parameter (N/m^2^), elastic modulus (Pa/kPa/MPa), apparent elastic modulus (Pa), complex modulus (kPa), or magnitude of complex shear modulus (kPa). ^d^ Viscoelasticity-related metrics, unit depends on the reported metric: Time- or frequency- dependent mechanical behavior combining elastic and viscous responses. In this table, viscoelasticity is reported as complex modulus (kPa), magnitude of complex shear modulus (kPa), viscoelastic ratio (dimensionless), phase angle (dimensionless); ^e^ Maximum elongation ratio, dimensionless: The maximum relative extension ratio achieved by tissue under loading; ^f^ Anisotropy, dimensionless or direction-specific modulus in Pa/kPa: direction-dependent mechanical behavior in which tissue properties differ along different fiber orientations; ^g^ Compliance, depending on the test: the extent of deformation or change produced by a given load. Higher compliance indicates greater deformability; ^h^ Stress relaxation, %: Time-dependent decrease in stress under a constant imposed strain; ^I^ Equilibrium stress, Pa/kPa: the steady-state stress reached after stress relaxation under a constant strain, reflecting passive resistance after transient viscoelastic effects have dissipated. 3DMMRE: Three-dimensional multi-frequency magnetic resonance elastography; IFEA: Inverse finite element modeling; AFM: Atomic force microscopy; PVE: Poro-viscoelastic model

**Table 2:** Overview of biomechanical cues, mechanosensors, and pregnancy outcomes

Biomechanical Cues	Mechanosensors	Mechanisms	Main Tissue/ Cell Type	Physiological Process	Pathological Associations	*Ref.*
Stiffness	Myosin-II	Myosin-II	STB	Trophoblast Fusion, Morphological, and Hormone release	Not reported	77
	Piezo1	Piezo1-Glycolysis-Ca^2+^-PKC -IL8/G-CSF	dESF	Trophoblast invasion	PAS	8
	Not reported	Not Reported	Early Embryo	Early embryo development	Embryo Arrest	56
	Fibrillin1	Feedback Positive	EVT	EVT invasion	Not Reported	66
	Not reported	LINC00458-SMAD2/3	hPSCs	Endodermal lineage differentiation	Not Reported	59
Shear Stress	Piezo1/YAP	Piezo1-Ca^2+^-YAP-PFKFB3 Piezo1-eNOS	EPCs	Glycolysis Vascularization	PE	154
	Piezo1 TRPV4 Glycocalyx	PGE2/PGI2-paracrine Piezo1-eNOS-NO TRPV4- Ca^2+^ Glycocalyx-NO	Endothelial	Decidualization Vasodilation	SGA PE	69, 84-86, 147-149
	Piezo1	Piezo1-Ca^2+^-TMEM16F cAMP-PKA-PIGF	Trophoblasts (STB)	Trophoblast fusion and migration Vascularization Metabolism	Embryo death PE	79, 89-91
	Focal adhesion	Focal adhesion assembly and actin polymerization	Fibroblasts Stroma cells	Pig placenta sculpt folds	Not reported	87
Tension	Not reported	IL-8/MCP3	Fibroblasts	Cervical Remodeling	Not reported	115
	Piezo1	Piezo1-Inflammatory	Myometrium	Uterine Contraction	PTB	166, 167
	Vinculin	Vinculin-occludin/ZO-1-ppMRLC/Myh9	Blastomeres	Embryo compaction ICM/TE specification	Not reported	53
Compression	Syndecan1	Syndecan1- E-cadherin	STB	Trophoblast fusion	Implantation failure	80
Traction force/ Contractility	YAP Actomyosin	Actomyosin /YAP	Blastomeres	Embryo Compaction ICM/TE Specification Implantation pattern	Embryo quality Implantation failure	51, 52, 61
Oscillation	YAP	PDGFRα/YAP	Early embryo	ICM formation	Not reported	54
ECM (Composition /Arrangement)	Adhesion molecule	Actin cytoskeleton	Trophoblast (CTB)	Endometrial-trophoblast adhesion Trophoblast invasion	RPL RIF	67, 75
	Collagen	Collagen-LAIR1 NKp30/Perforin Type I/V collagen	Macrophage dNK Th17/Treg	Immune tolerance	RPL	97, 98, 127, 131
	α-actinin-1	ACTN1-NEBL-F-actin	Epithelial	Endometrial receptivity	RIF	124
	Collagen	SAA1-TypeI collagen	Fetal Membrane	Rupture of fetal membrane	PPROM	160
	Versican	Not reported	Stromal cells	Spiral artery dilation	Not reported	99

Note: The biomechanical terms listed in this table refer to biomechanical cues. Stiffness mainly refers to ECM or substrate stiffness. Shear Stress mainly refers to blood flow-induced wall shear stress acting on cells. Tension refers to tensile forces generated between neighboring cells or across cell-cell junctions. Compression refers to compressive forces generated between adjacent cells or within confined tissue spaces. Traction force refers to the force exerted by cells on the adhesive substrate. Contractility refers to active force generation by cells, often mediated by actomyosin activity. Oscillation refers here mainly to blastocyst cavity oscillation or periodic biomechanical fluctuation during early embryo development. STB: syncytiotrophoblast; PAS: placenta accreta spectrum; dESF: decidual endometrial stroma fibroblasts; EVT: extravillous trophoblast; EPCs: Endothelial progenitor cells; hPSCs: human pluripotent stem cells; SGA: small for gestational age; PE: preeclampsia; RPL: recurrent pregnancy loss; RIF: recurrent implantation failure; CTB: Cytotrophoblast; PGE2: Prostaglandin E2; PGI2: Prostacyclin PGI2; ICM: Inner cell mass; TE: trophectoderm; PPROM: preterm premature rupture of membranes; ECM: Extracellular Matrix.

## Abbreviations

ECM: Extracellular Matrix
FAK: Focal Adhesion Kinase
ICM: Inner Cell Mass
TE: Trophectoderm
ENaC: Epithelial Sodium Channels
dS: Decidual Stromal Cells
CTB: Cytotrophoblast
STB: Syncytiotrophoblast
EVT: Extravillous Trophoblast
enEVT: Endovascular Extravillous Trophoblast
iEVT: Interstitial Extravillous Trophoblast
FGR: Fetal Growth Restriction
dNK: decidual natural killer
RPL: Recurrent pregnancy loss
CSP: Cesarean Scar pregnancy
PAS: Placental accrete spectrum
ROS: reactive oxygen species
PE: Preeclampsia
NO: nitric oxide
PPROM: preterm premature rupture of membranes
CI: cervical insufficiency
EPCs: endothelial progenitor cells
PTB: Preterm birth
AFM: Atomic force microscopy
